# Breeding Potential for Increasing Carbon Sequestration via Rhizomatous Grain Sorghum

**DOI:** 10.3390/plants14050713

**Published:** 2025-02-26

**Authors:** Yaojie Zheng, Dirk B. Hays, Russell W. Jessup, Bo Zhang

**Affiliations:** 1 School of Plant and Environmental Sciences, Virginia Tech, Blacksburg, VA 24061, USA; yaojie@vt.edu; 2 Texas A&M AgriLife Research and Extension Center at Weslaco, Weslaco, TX 78596, USA; dirk.hays@ag.tamu.edu; 3 Department of Soil and Crop Sciences, Texas A&M University, College Station, TX 77843, USA; rjessup@tamu.edu

**Keywords:** rhizomatous sorghum, carbon sequestration, source and sink, crop improvement

## Abstract

Rhizomes, key carbon sequestration sinks in perennial crops, are hypothesized to exhibit a trade-off with grain yield. This study evaluated rhizomatous grain sorghum populations for increasing carbon sequestration potential. Twelve F_3:4_ heterogeneous inbred families (HIFs) from a *Sorghum bicolor* (L.) Moench × *Sorghum propinquum* (Kunth) Hitchc cross were tested in a greenhouse, and two F_4:5_ HIF progenies were field tested. Traits measured included rhizome biomass, root biomass, total belowground biomass, and grain yield. Rhizome biomass showed high heritability (0.723) and correlated strongly with belowground biomass (*r*_1_ = 0.95; *r*_2_ = 0.97) in both F_4:5_ HIFs, suggesting the potential of rhizomes to sequester carbon. Contrary to the hypothesized trade-off, a positive relationship between rhizome biomass and grain yield was observed, potentially via rhizome-derived shoots, and individual plants pyramiding high rhizome biomass, biomass yield, and grain yield were also identified. Using bulked segregant analysis (BSA), twenty simple sequence repeat (SSR) markers linked to eight genomic regions associated with rhizome presence were identified, with five regions potentially being novel. This study suggests that breeding rhizomatous grain sorghum with high rhizome biomass could enhance carbon sequestration while preserving agronomic yields, offering new insights for future breeding and mapping initiatives.

## 1. Introduction

The greenhouse effect driven by greenhouse gases (GHGs) has become an escalating concern. As of November 2023, atmospheric carbon dioxide, a primary GHG, reached a concentration of 420.15 parts per million (ppm), marking a 6% increase from 2013, and is continuing to rise at an annual rate of 2.16 ppm [1]. Mitigating this trend involves enhancing plant-based atmospheric carbon absorption and stable belowground storage, a process known as carbon sequestration [2].

Rhizomes, major carbon sinks in plants, are capable of significantly enhancing the carbon sequestration potential of widely grown grain crops [3]. As modified belowground stems found in perennial plants, rhizomes differ from the roots of annual crops based on their functions in resource storage and vegetative reproduction. Their carbon storage capacity is particularly noteworthy. For example, in C4 perennial grass species like *Miscanthus sinensis Anderss*., *Miscanthus* × *giganteus*, and *Arundo donax* L., rhizome biomass yields can reach up to 19.5 mg ha^−1^. This translates to a carbon storage of approximately 7.8 mg ha^−1^, substantially (7.4 mg ha^−1^) higher than the carbon content in annual sorghum roots [4,5,6,7]. Therefore, the capacity of rhizomes to sequester carbon depends on the ratio of their biomass to root biomass. The carbon sequestration of rhizomatous crops will be maximized if rhizome biomass dominates total belowground biomass.

Sorghum is a key crop globally, with diverse cultivations in grain and forage production [8]. Breeding efforts primarily focus on high-grain-yield varieties in regions where sorghum grains serve as a staple in human and animal diets. In countries like the United States, where sorghum is predominantly an animal feed, varieties yielding high grain and substantial aboveground biomass are of interest [9]. Currently, a majority of the annual sorghum yield is made up of cultivated grain sorghum species. However, within the *Sorghum* genus, perennial species such as *Sorghum propinquum* (Kunth) Hitchc (2n = 2x = 20) and *Sorghum halepense* (L.) Pers. (2n = 2x = 40) exist, both featuring rhizomatous genotypes in their genetic makeup. Notably, *Sorghum propinquum* shares the same ploidy level with annual grain sorghum [*Sorghum bicolor* (L.) Moench (2n = 2x = 20)], rendering it an ideal candidate to develop new perennial grain sorghum varieties [10].

Genetic research on rhizome development has evolved over decades, identifying key genetic loci and molecular markers for traits such as overwintering and rhizome number [11,12,13,14,15]. Many quantitative trait loci (QTLs) regulating rhizome traits are evolutionarily conserved across species. For example, in rice (*Oryza sativa* L. × *Oryza longistaminata* A. Chev. & Roehr.), three QTLs correspond to sorghum homologs distributed among chromosomes 4, 6, and 10 [16,17]. Similar parallels exist in maize (*Zea mays* ssp. *parviglumis* × *Zea diploperennis*), although the number of homologous QTLs is fewer due to greater genetic distances between species [18]. Rhizomatousness encompasses various aspects, including the number of rhizome-derived shoots, rhizome length, and the count of subterranean rhizomes [14]. However, research specifically addressing rhizome biomass in sorghum, a direct measure of carbon sequestration in perennial crops, remains limited.

Currently, a major barrier to replacing perennial grain crops with annuals is the widely held yet unproven belief in a trade-off, where grain yield in perennials is thought to decrease due to less photoassimilate being allocated to grain production [19,20]. However, this trade-off is highly dependent on the environment and not a physiological fact. In resource-limited environments, perennials prioritize rhizome development for rapid vegetative reproduction and space competition. This allocation can shift in resource-rich agricultural settings [21]. Physiologically, the negative trade-off might occur if carbon assimilation primarily originates from the main stem and both grains and rhizomes act as carbon sinks. While rhizomes generally grow horizontally underground, they can also become rhizome-derived shoots (RDSs) that are relatively self-sufficient, capable of independent root and rhizome development, photosynthesis, and grain production [22,23]. Consequently, a strict trade-off between resources allocated to rhizomes and grains may not be necessary. Moreover, rhizome longevity in perennials allows them to store more carbon than seeds, facilitating earlier spring growth and extending the photosynthetic period [21]. The increased photosynthetic output from autotrophic RDSs and an extended growing season could balance the resources invested in rhizome development. Model predictions even suggest that perennial grain yields could match those of annuals with increased biomass [24]. Identifying ways to circumvent this trade-off could enhance the viability of introducing rhizomatous traits into widely cultivated annual cereals, offering a more effective approach to carbon sequestration.

Beyond grain yield, biomass is another agronomically important yield-related trait. The connection between rhizomatousness and aboveground biomass involves several traits underpinned by two physiological principles. Firstly, both rhizomes and basal tillers originate from axillary buds on the bottom shoot nodes [13]. Secondly, the apical meristems of rhizomes and axillary buds on rhizome nodes can develop into aerial shoots (rhizome-derived shoots) [25]. Given their shared developmental origin, the genetic loci influencing rhizomes and basal tillers partially overlap. In perennial sorghum (*S. bicolor* × *S. propinquum*), this overlap includes five QTL regions affecting rhizomatousness and vegetative tillering, including four genes involved in branching [13,26,27]. However, despite genetic similarities, rhizomes and tillers are morphologically and functionally distinct, exhibiting different gene expressions in their differentiation [28]. This genomic overlap forms the genetic basis for the correlation between rhizomes and basal tillers. However, their differentiation and the growth direction of rhizomes determine their dynamic relationships with aboveground biomass.

In studies investigating regrowth, underground organs of rhizomatous sorghum are typically left in the soil, with regrowth measured by counting rhizome-derived shoots [13,14,15,29]. This approach, however, does not measure rhizome biomass, a direct indicator of carbon sequestration, and leaves the corresponding genetic loci unidentified. In our study, we excavated rhizomes to accurately assess rhizome biomass. The breeding potential based on ecological benefits was evaluated by direct trait performance, analysis of variance (ANOVA), and correlation analysis. Additionally, developing molecular markers and defining genetic regions, another novelty in this study, will aid molecular breeding and future genetic mapping research.

## 2. Results

### 2.1. Basic Statistics and Genetic Pattern

Basic statistical parameters and Shapiro–Wilk normality test results for both belowground and aboveground traits are presented in Table 1 for the two F_4:5_ heterogeneous inbred families derived from the F_3:4_ generation and in Table S1 for the F_3:4_ generation. The two HIFs demonstrated a broad phenotypic range in rhizome biomass, with average weights of 20.26 g and 26.13 g for the low- and high-rhizome-biomass families, respectively. This equals carbon sequestration of 14.86 g and 19.16 g if 50% dry weight is assumed to be carbon-containing photoassimilates [30]. Notably, rhizome biomass averages exceeded those of fibrous root biomass in both HIFs. However, the F_3:4_ generation, which comprised 41 plants across seven HIFs, exhibited a marked decrease in rhizome biomass compared to the F_4:5_ generation. This reduction may be attributable to the limited belowground space in greenhouse pots, which could hinder rhizome growth, as observed in tall fescue (*Festuca arundinacea* Schreb.) by De Battista and Bouton [31]. The F_3:4_ root and belowground biomass averages were both intermediate between BTx623 and *S. propinquum*, while the F_4:5_ generation showed a lower root biomass average, possibly due to enhanced rhizome extension and the corresponding photoassimilate allocation in field troughs. In addition, *S. propinquum,* as a short-day parent, failed to flower or set grain under supplemental lighting in greenhouse conditions, unlike BTx623 and all F_3:4_ progenies from the seven families.

Trait distributions were unimodal and bell-shaped in both HIFs, suggesting their possible regulation by quantitative loci (Table 1). The Shapiro–Wilk test was conducted on each trait, and the results indicated that except for rhizome length and aboveground biomass, all traits deviated from normal distribution. Root biomass was normally distributed in the low-rhizome-biomass family but not in the high-rhizome-biomass family, which could be due to variable plant numbers in each family and missing data. Attempts to normalize distributions through logarithmic, arctangent, and reciprocal transformations were unsuccessful (Table S2). The possible reason for this lack of normality could be explained by the selection of extreme F_4:5_ HIFs from the F_3:4_ generations, which may have reduced heterozygosity of loci, therefore leading to diminished genetic segregation in the F_4:5_ generation. Moreover, if the majority of loci exhibit additive or partially dominant effects, this would result in fewer individuals with intermediate trait performance, further contributing to the observed skewness.

### 2.2. Variation Analysis and Heritability Estimation

The ANOVA was constructed for every trait in F_4:5_ based on a randomized complete block design (RCBD), as detailed in Table 2. For traits not conforming to normal distribution, the nonparametric Friedman test was applied instead of the traditional Fisher’s F test. Broad-sense heritability estimates are also included in Table 2. Results indicated no significant replication effects for any trait; however, these effects accounted for a large part of variance in a few traits, including rhizome number (21.51%), rhizome length (62.27%), and aboveground biomass (44.56%). Conversely, family effects were significant for rhizome biomass, root biomass, belowground biomass, growth angles, tiller number, shoot number, and plant height, contributing from 82.93% to 96.30% of the total phenotypic variance. These results suggest significant differences between the two families for these traits, and with heritability values ranging from 0.72 to 0.93, they appear to be selectable for breeding improvement.

In contrast, rhizome number, rhizome length, flowering time, aboveground biomass, and grain yield had insignificant family effects, and the heritability of flowering time was also low. The heritability could not be calculated in rhizome number, rhizome length, aboveground biomass, and grain yield because the genetic variance was lower than the error variance. The main possible explanation for this is that those four traits were more likely affected by the environment–genotype interaction, which masked the extremely low estimation of genotypic variance.

### 2.3. Correlation Analysis

To investigate the relationships among traits, Pearson’s correlation analysis was applied to continuously distributed traits in F_4:5_ and F_3:4_ HIFs, while Spearman’s correlation was utilized for discrete traits such as rhizome number, flowering time, basal tiller number, and rhizome-derived shoot number.

**Figure 1:**
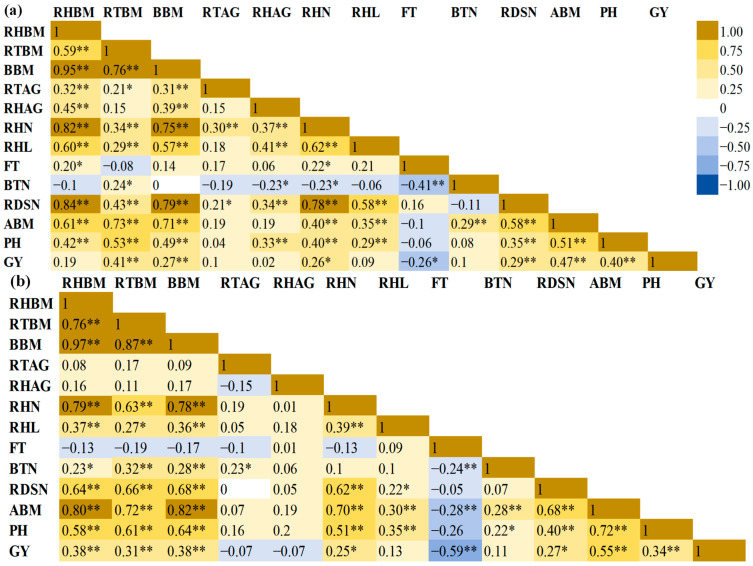
Correlation heatmap of (**a**) low-rhizome-biomass family and (**b**) high-rhizome-biomass family. Numbers in the box are correlation coefficients (*r*). * Significant at the 0.05 probability level. ** Significant at the 0.01 level. RHBM: rhizome biomass; RTBM: root biomass; BBM: belowground biomass; RHAG: rhizome angle; RTAG: root angle; RHN: rhizome number: RHL: rhizome length; FT: flowering time; BTN: basal tiller number; RDSN: rhizome-derived shoot number; PH: plant height; ABM: aboveground biomass; GY: grain yield.

In the F_3:4_ generation, no significant correlation was found between rhizome biomass (RHBM) and belowground biomass (BBM) (Table S3). In contrast, root biomass (RTBM) showed a strong positive correlation with BBM (*r* = 0.99), likely due to limited rhizome proliferation under controlled environments in greenhouses, making RTBM the primary contributor to BBM. The dynamic shifted in the F_4:5_ generation (Figure 1), where RHBM exhibited a strong positive correlation with BBM in both families (low-rhizome-biomass family *r* = 0.95; high-rhizome-biomass family *r* = 0.97), signifying its major contribution to total BBM. RTBM remained significantly correlated to BBM but as a secondary contributor. Additionally, significant positive correlations were observed in F_4:5_ between RHBM and other rhizome-related traits as well as aboveground traits. These correlations suggest a synergistic growth pattern in this sorghum population, with RHBM playing a decisive role in BBM. When rhizomes initiate, they tend to grow upward as aerial shoots rather than expanding extensively belowground, as seen in some rhizomatous sorghum species. This growth pattern of rhizome-derived shoots then positively contributes to aboveground biomass and grain yield, a trend also supported by the positive correlations between rhizome number and rhizome-derived shoot number as well as aboveground biomass and grain yield.

Fibrous root biomass (RTBM) maintained its essential role in constituting belowground biomass after rhizome biomass, showing positive correlations with rhizome biomass (low-rhizome-biomass family *r* = 0.59; high-rhizome-biomass family *r* = 0.76). Significant correlations were observed for both RTBM and BBM with rhizome number, length, rhizome-derived shoot number, aboveground biomass, plant height, and grain yield. This suggests that a robust fibrous root system is important in both subterranean and aerial biomass as well as grain production. For root and rhizome growth angles, only the low-rhizome-biomass family showed positive correlations with belowground traits. In contrast, the high-rhizome-biomass family displayed no significant correlations, likely due to genetic differentiation and environmental interactions.

For aboveground traits, rhizome-derived shoot number, biomass, plant height, and grain yield were all significantly interrelated. However, basal tiller number (BTN) exhibited weak or no significant correlations with other aerial traits, possibly because its function has been overshadowed by rhizome-derived shoots, another form of aerial shoot. The relationship between flowering time and basal tiller number and grain yield was inversely correlated, suggesting that earlier flowering may lead to an earlier cessation of apical dominance and a longer period for grain development and filling. Flowering time, however, was not associated with rhizome-derived shoot number, potentially because the rhizome apical meristem is independently regulated by its own auxin source.

### 2.4. Genomic Evaluation Results

**Figure 2:**
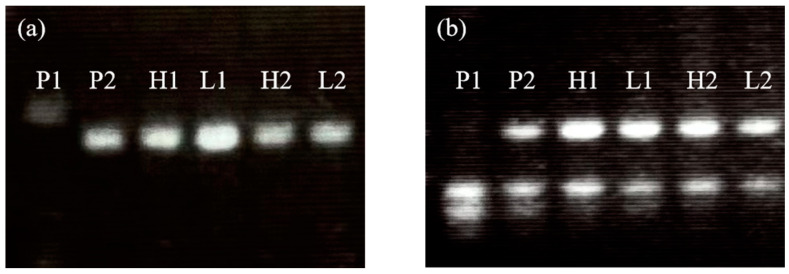
The selected gel figure of the markers linked to rhizome presence with co-dominant type (**a**) and hemizygous type (**b**). P1: *S. bicolor*; P2: *S. propinquum*; H1: first-round high-rhizome-biomass bulk; L1: first-round low-rhizome-biomass bulk; H2: second-round high-rhizome-biomass bulk; L2: second-round low-rhizome-biomass bulk.

The individual rhizome biomass values for two-round BSA pool participants are shown in Table 3. Out of 259 SSR markers screened, 53 (20.46%) displayed polymorphism between the parental lines, including two distinct types (Figure 2). Contrary to our initial hypothesis, the BSA pools did not reveal markers unique to the high-rhizome-biomass pool; instead, up to 20 markers displayed the same genotype between high- and low-rhizome-biomass pools but consistent with the *S. propinquum*-specific band (Figure 2, P2). Despite clear phenotypic divergence, both pools consisted of individuals capable of rhizome development, which is distinct from *S. bicolor* (Figure 2, P1). This suggests that rhizome biomass may not be governed by a single locus or a few loci with large effects, limiting the discriminative power of phenotype-based BSA pools. The 20 identified markers (Table S4), while not exclusively associated with rhizome biomass, are linked to the presence or absence of rhizomes and may serve as molecular markers for this trait.

The identified markers were distributed across eight genomic regions on sorghum chromosomes 1, 2, 4, 5, 7, 8, 9, and 10 (Table 4), supporting the quantitative nature of rhizomatousness. Notably, the region on chromosome 1 (57.3~80.5 Mb) overlapped with several known QTLs, namely qRZ1.2 and Ln2010RDS, which regulate the presence and number of rhizome-derived shoots, respectively [13,15]; Xcup73-Xcup22, Ln2010Dist, and Ln2011Dist, associated with rhizome distance [13,15]; qRN1.2, linked to rhizome number [13]; and over-wintering2011B, related to overwintering [15]. Additionally, regions on chromosomes 4 and 7 also overlapped with previously reported QTLs for rhizome traits, including rhizome-derived shoot number (qRZ7.1), rhizome number (qRN7.1) [15], and regrowth [14]. Four of these regions also intersected with vegetative branching QTLs, including basal tiller and axillary branch number [32].

Aside from these overlaps, five potentially novel genomic regions were identified in this study. Most regions were no longer than 6.3 Mb, except for a single marker on chromosome 10 and a larger region on chromosome 2 (11.8 Mb) (Table 4). These target QTLs are likely located within or flanking these intervals, and larger regions may encompass multiple loci of interest.

## 3. Discussion

Rhizomes represent an ecologically beneficial organ for atmospheric carbon dioxide sequestration, potentially mitigating the greenhouse effect. Rhizome biomass directly reflects carbon sequestration capacity, as 40–50% of rhizome dry matter consists of photosynthetically derived carbohydrates [30]. In our F_4:5_ generation’s high-rhizome-biomass family, the average rhizome biomass was 26.13 g per plant, with the highest at 82.47 g. This translates to a potential yield of 6.11 mg ha^−1^ dry rhizomes, equivalent to sequestering 4.59 mg ha^−1^ of carbon dioxide, under the planting density used in this study (74,131 plants ha^−1^). This suggests that converting all current sorghum croplands to our perennial sorghum line could offset the annual carbon emissions of approximately 742,000 Americans in one season [34].

Studying the feasibility of developing high-rhizome-biomass varieties is one of the objectives of this study. Although no prior studies have focused on rhizome biomass heritability in sorghum or other C4 grasses, related traits have been examined in johnsongrass with lower heritabilities, namely rhizome number (0.077) and rhizomatousness (0.34) [14,35]. In contrast, the high heritability (0.723) observed in our study indicates strong breeding selectability. This disparity may be caused by genetic differences between species and cultivation conditions. Johnsongrass has more and longer rhizomes compared to *S. propinquum* [36,37], and field conditions were also different in our field cultivation, which followed standard sorghum agronomy practices. Therefore, adequate nutrition and water greatly alleviate intraspecies competition, which may weaken the environmental impact.

In the United States, sorghum is primarily used for livestock silage and bioenergy fuel production, with both applications dependent on vegetative aboveground biomass. In our F_4:5_ population, the plant with the highest aboveground biomass (250.67 g) suggested a potential yield of 18.58 mg ha^−1^ in aboveground dry weight. Notably, a significant positive correlation existed between aboveground and rhizome biomass (*r* = 0.61 in the low-rhizome-biomass HIF and *r* = 0.80 in the high-rhizome-biomass HIF), indicating concurrent potential for enhancing both belowground and aboveground biomass accumulation. The plant with the highest aboveground biomass also exhibited high rhizome biomass (57.07 g), nearly two standard deviations above the family mean. When considering total biomass (above and belowground combined), our population could potentially yield up to 22.81 mg ha^−1^ of dry matter, making it competitive in the current forage market.

**Figure 3:**
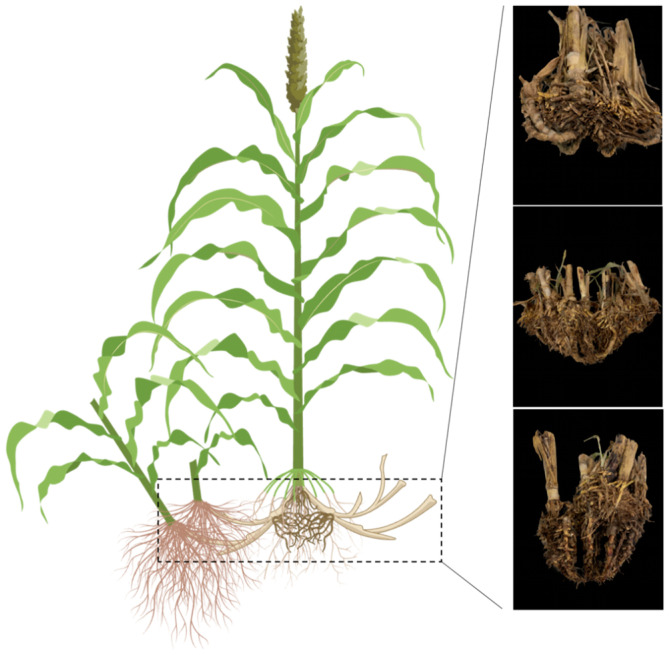
Schematic graph and individual samples represent the developing pattern of rhizomes in the F_4:5_ population (created with BioRender.com).

The relationship between rhizome biomass and total aboveground biomass exhibits strong plasticity, influenced by various mechanisms. Firstly, this relationship may be governed by the developmental fate of basal axillary buds, which give rise to both rhizomes and basal tillers. The differentiation of these buds into basal tillers or rhizomes can significantly impact aboveground biomass [13,17]. Factors like nitrogen availability, daily temperature, and photoperiod can influence this differentiation, with responses varying across species. For instance, higher temperatures and longer daylight hours stimulate rhizome formation in Kentucky bluegrass (*Poa pratensis* L. Ecotypes) [38,39], but the reverse is true in quackgrass (*Agropyron refiens* L. Be) [40]. Our study proposes another mechanism, where rhizomes, rather than extending horizontally, bend upwards as aerial shoots, contributing essentially to aboveground biomass (Figure 3). This correlation between aboveground biomass and stem count, including tillers and rhizome-derived shoots, aligns with findings from other perennial sorghum studies [29,41,42]. Interestingly, while basal tiller number correlates significantly with aboveground biomass, its correlation coefficient is lower than that of rhizome-derived shoot number, diverging from the findings in Kong et al. [32].

Another major role of sorghum as a grain for humans and animals has traditionally been filled by annual species, with limited progress in the cultivation of perennial varieties. This stagnation is partly due to the purported ‘trade-off’ between resource allocation to grains and belowground organs, suggesting rhizome development hinders grain filling [20,23,43]. This concept is based on the assumption that the photosynthate ‘source’ is fixed and that rhizomes and grains compete for the same photoassimilates. However, our study found no negative impact of rhizome biomass on grain yield in either the F_3:4_ or F_4:5_ generations. In fact, within the high RHBM family, yield was positively correlated with RHBM, implying that an enhanced rhizome system may contribute to higher yield. Conversely, within the low RHBM family, yield was not correlated with RHBM. Rhizomes enhanced grain yield by producing numerous autotrophic rhizome-derived shoots (RDS), which formed their own inflorescences and contributed to photoassimilate production (Figure 3) [22,44]. This finding challenges the traditional ‘trade-off’ notion and aligns with Habyarimana et al. [29], who also reported a positive correlation between stem number, rhizome development, and grain yield in a *S. bicolor* × *S. halepense* population. Interestingly, second-year yields from rhizome-derived shoots (regrowth) were comparable to first-year yields [45], suggesting their yielding potential for grain production. Further research is needed to compare the grain yield of first-year RDS with the main shoot yield. Moreover, management techniques like mowing or clipping can synchronize flowering between the crown and RDS, ensuring uniform growth stages.

Given that rhizome development does not adversely affect grain yield, individuals combining both high grain yield and high rhizome biomass may exist. In our F_4:5_ population, the highest grain yield (46.70 g) was observed in a low-rhizome-biomass HIF individual, which also produced a notable 22.06 g of rhizome biomass. This indicates the potential for our population to yield up to 1.6 Mg ha^−1^ of dry rhizomes alongside 3.5 mg ha^−1^ of grains.

The bulked segregant analysis (BSA) offers a practical method for marker linkage analysis [46]. However, our findings suggest that the traits under study may not be controlled by a few large-effect loci, which diminishes the efficacy of BSA pools for screening purposes. This limitation introduces greater challenges for genetic mapping that requires higher resolution. With the advancement of genotyping by sequencing (GBS) technology, either high-density single-nucleotide polymorphism (SNP) markers for linkage mapping or genome-wide association studies (GWAS) are expected in future research.

## 4. Materials and Methods

### 4.1. Greenhouse Cultivation

The greenhouse study utilized twelve rhizomatous F_3:4_ heterogeneous inbred families (HIFs) from a cross between *Sorghum bicolor* (BTx623) × *Sorghum propinquum* (unnamed line), as described by Paterson et al. [14]. These HIFs and two parental lines, used as controls, were sown on 23 September 2019 at the greenhouse of the Institute for Plant Genomics and Biotechnology, Texas A&M University. The greenhouse used natural sunlight and sodium halide lights for lighting supply. Ten seedlings each of HIF and parental lines were selected, and individual plants were planted in a two-gallon pot filled with a growth mixture (Jolly Gardener^®^, PRO-LINE, C/20 Growing Mix; Old Castle Lawn & Garden, Atlanta, GA, USA). The arrangement followed a randomized complete block design. The seedlings received irrigation every four days before the boot stage, which increased to every two days in the post-boot stage. Fertilization was conducted weekly with Peters Professional^®^ 20–20–20 General Purpose water-soluble fertilizer (JR Peters Inc., Allentown, PA, USA) at a concentration of approximately 5.1 g L^−1^, which was stopped after the onset of the boot stage. Finally, 41 plants distributed among seven HIFs survived.

### 4.2. Field Cultivation

Following the harvest of the F_3:4_ generation, two F_4:5_ progeny lines, representing the highest and lowest rhizome biomass, were chosen from one F_3:4_ HIF. On 12 May 2020, 110 F_4:5_ seeds from each selected line were germinated in seedling trays within the IPGB greenhouse. Upon most seedlings reaching the five-leaf stage, they were transplanted to a field trough at the Texas A&M University Farm (30°31′49.3″ N, 96°25′15.4″ W) at a density of 74,131 plants per hectare. The plot was filled with fine sand (fine, smectitic, thermic Udic Paleustalfs), which is beneficial for rhizome development and makes it easier to dig out and wash them. The planting method also followed a randomized complete block design with two replications, one row of each. The field trough received weekly irrigation and fertilization with Miracle-Gro^®^ Water-Soluble All-Purpose Plant Food (Scotts Miracle-Gro Co., Marysville, OH, USA) at a concentration of 5.1 g L^−1^. A total of 107 plants from the low-rhizome-biomass family and 88 from high-rhizome-biomass family finally survived for data collection.

### 4.3. Phenotypic Data Collection

For the F_3:4_ population, mature plant heads from each stem and tiller were harvested and threshed. The seeds from each plant were measured for total weight with 0.01 g precision to determine grain yield (GY), expressed as weight per plant (g plant^−1^). Belowground traits were assessed by carefully excavating each plant from the pot with its soil, followed by a high-pressure water wash to remove the soil. The aboveground parts were removed, and the belowground portions were air-dried. Rhizomes were then separated from fibrous roots to determine rhizome biomass (RHBM) and root biomass (RTBM), with total belowground biomass (BBM) calculated by summing RHBM and RTBM.

**Figure 4:**
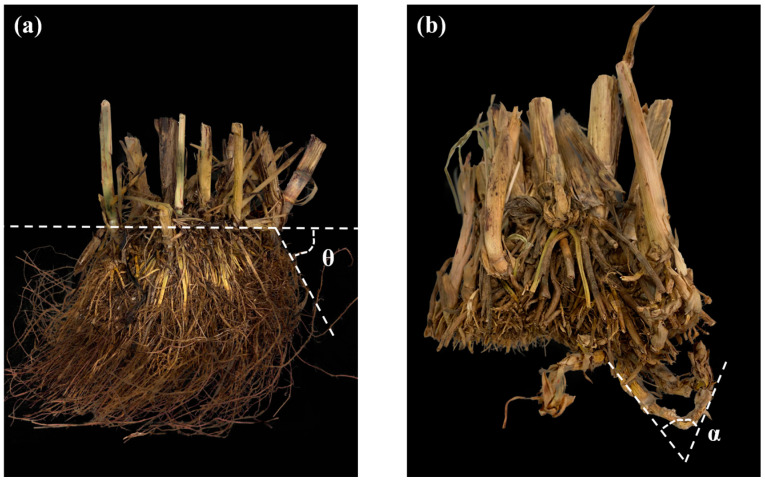
Ways to measure the root growth angle θ (**a**), and rhizome bending angle α (**b**).

In the field trough study of two F_4:5_ HIFs, flowering time (FT) was recorded for each plant, with observations made weekly from 29 July 2020, when flowering commenced. The final harvest was on 28 October 2020, and the grain yield (GY) was measured the same way as the F_3:4_. Plant growth was terminated on 17 November 2020. Aboveground parts were harvested at ground level to measure plant height (PH) to the nearest 0.1 cm, followed by oven-drying at 55 °C for 24 h to determine aboveground biomass (ABM) with a precision of 0.01 g. Belowground organs were extracted with a 40 × 40 × 40 cm soil core and processed as with the F_3:4_, including oven-drying at 55 °C for 72 h. Subsequently, fibrous roots were separated for RTBM measurement, exposing the rhizomes for basal tiller number (BTN) and rhizome-derived shoot number (RDSN) counts. Rhizome length (RHL) was measured to 0.1 cm precision, with the rhizome number (RHN) being counted. Nodal root angle (RTAG) and rhizome angle (RHAG) were determined as illustrated in Figure 4, with an accuracy of 1°. Rhizome biomass (RHBM) and belowground biomass (BBM) were quantified similarly to the F_3:4_.

### 4.4. Statistical Analysis

For the thirteen traits investigated in this study, their distribution was assessed in JMP^®^ Pro 15.0.0 (390308) (SAS Institute Inc., Cary, NC, USA) by plotting histogram graphs with normal fit curves. Descriptive statistics, including mean, standard deviation, and range, characterized the basic properties of each trait. The Shapiro–Wilk test, performed using the Proc Univariate Normal function in SAS^®^ [SAS (r) 9.4 (9.04.01M2P072314)] (SAS Institute Inc., Cary, NC, USA), provided a more precise evaluation of normality. Analysis of variance (ANOVA) for family means was executed in JMP Pro 15.0.0 to compare traits. The heritabilities were calculated subsequently and considered as broad-sense heritabilities, as the dominant variance still existed in the F_4:5_ selfing generation. The formula is as follows:

σ_G_^2^ = (σ_F_^2^ − σ_e_^2^)/r (1)

H^2^ = σ_G_^2^/(σ_G_^2^ + σ_e_^2^) (2)

where σ_F_^2^ is the variance between families; r is replication; σ_G_^2^ is the genotypic variance; σ_e_^2^ is the error variance; and H^2^ is heritability.

The Pearson correlation coefficient (*r*) was computed for each pair of continuously distributed traits using the Proc Corr function in SAS. For the discrete traits, including rhizome number (RHN), flowering time (FT), basal tiller number (BTN), and rhizome-derived shoot number (RDSN), Spearman’s rank correlation coefficient (ρ) was utilized in place of Pearson’s *r*. Results for the F_4:5_ generation are presented on a family basis.

### 4.5. Genomic Evaluation

DNA extractions were performed on the parental lines and two F_4:5_ HIFs. Young leaf tissue was harvested at the five-leaf stage and immediately stored at −80 °C. Approximately 100 mg of the freeze-dried tissue was cut and placed into a 2 mL microtube. The extraction followed the standard CTAB protocol [47] with two modifications. DNA was homogenized using stainless steel beads in a FastPrep-96™ high-throughput bead-beating grinder (MP Biomedicals, Santa Ana, CA, USA), and extraction buffer containing 63.77 g L^−1^ sorbitol, 12.1 g L^−1^ Tris, and 1.68 g L^−1^ EDTA was added before grinding. Genomic DNA integrity was verified via 1% agarose gel electrophoresis, and DNA concentration was quantified using a Genesys™ 10 UV spectrophotometer (Thermo Fisher Scientific, Waltham, MA, USA).

For bulked segregant analysis (BSA), DNA from the five individuals with the highest and lowest rhizome biomass was pooled. The DNA of each plant was equally mixed and diluted to a final concentration of 10 ng μL^−1^. The second-round BSA pools increased DNA from twelve individuals per extreme pool. We utilized 259 SSR markers (sourced from Winn et al. [48]) to analyze genetic polymorphism between the parents. PCR amplifications were performed in a 20 μL reaction system containing 2 μL of 10 × Taq buffer, 0.5 μL of 4 mM dNTPs, 1 μL of 25mM MgCl_2_, 4 μL of 10 ng μL^−1^ DNA, 0.2 μL of 5 U mL^−1^ Taq Polymerase, 6.3 μL of ddH_2_O, and 3 μL each of 2μM forward and reverse primers. The PCR conditions followed those outlined by Winn et al. [48], with an increase to 50 cycles for the annealing–extension step. PCR products were analyzed on a 3% agarose gel to identify polymorphisms between the parental lines. Polymorphic markers were then screened across the four BSA bulks to preliminarily identify genetic regions linked to the traits of interest.

## 5. Conclusions

In this study, we demonstrated the significant carbon sequestration potential of rhizomes, as evidenced by their biomass. Rhizomes were the primary contributors to total belowground biomass, and enhancing their biomass positively impacted various underground traits. Additionally, rhizomes were observed to bolster aboveground vegetative growth and grain yield by producing rhizome-derived shoots. The presence of individual plants with high rhizome and aboveground biomass, coupled with robust grain yield, suggests that breeding varieties with high rhizome biomass and shoot numbers could offer ecological, agricultural, and practical benefits.

Our findings also indicate that rhizome biomass, likely being a highly quantitative trait, limits the effectiveness of bulked segregant analysis (BSA) for identifying linked molecular markers. However, BSA remains useful for selecting markers associated with the presence or absence of rhizomes, a trait closely related to rhizome biomass. We identified twenty markers correlated to rhizome presence, defining eight genomic regions, five of which may be novel based on comparisons with prior studies. Future efforts should focus on genetic mapping of rhizome biomass using denser markers or high-throughput genotyping techniques.

## Figures and Tables

**Table 1:** The basic statistics of thirteen traits in F_4:5_ HIFs derived from the cross between BTx623 × *Sorghum propinquum*.

Traits ^2^	Basic Statistics				Test for Normality	
	Mean	SD	Minimum	Maximum	W-Value	Pr < W
RHBM (g)	20.26 ^1^	16.56	0.03	77.91	0.8907	<0.0001
	26.13 ^1^	17.27	0.46	82.47	0.9516	0.0040
RTBM (g)	12.62	5.43	0.93	26.76	0.9878	0.5490
	17.43	7.92	1.77	55.86	0.9025	<0.0001
BBM (g)	32.89	20.05	2.2	97.3	0.9337	0.0001
	43.25	24.05	2.65	125.72	0.9547	0.0057
RHAG (°)	36.00	16.33	10	120	0.8244	<0.0001
	53.57	26.06	15	120	0.8912	<0.0001
RTAG (°)	31.26	5.28	10	42.5	0.9388	0.0003
	34.00	5.24	25	55	0.9052	<0.0001
RHN (no.)	16.13	8.81	2	37	0.9684	0.0239
	16.20	7.62	1	40	0.9639	0.0250
RHL (cm)	4.85	1.90	0	9.9	0.9774	0.1365
	4.57	1.82	0.9	10.3	0.9718	0.0848
FT (d)	82.92	7.88	73	122	0.8226	<0.0001
	84.26	7.02	73	108	0.8476	<0.0001
BTN (no.)	4.21	2.42	1	12	0.9171	<0.0001
	2.73	1.75	1	9	0.8291	<0.0001
RDSN (no.)	17.10	11.21	0	44	0.9415	0.0004
	10.60	8.15	0	36	0.9081	<0.0001
PH (cm)	69.16	13.68	30.3	94.2	0.9685	0.0266
	81.19	18.75	15.3	114.6	0.9451	0.0017
ABM (g)	97.56	43.66	6.42	200.06	0.9778	0.1143
	100.37	49.93	9.63	250.67	0.9794	0.2126
GY (g)	7.75	7.36	0.14	46.70	0.7663	<0.0001
	6.73	5.74	0.16	31.58	0.8710	<0.0001

^1^ The value in the upper-half box is the low-rhizome-biomass family, and the value in the lower-half box is the high-rhizome-biomass family.^2^ RHBM: rhizome biomass; RTBM: root biomass; BBM: belowground biomass; RHAG: rhizome angle; RTAG: root angle; RHN: rhizome number: RHL: rhizome length; FT: flowering time; BTN: basal tiller number; RDSN: rhizome-derived shoot number; PH: plant height; ABM: aboveground biomass; GY: grain yield.

**Table 2:** ANOVA, variance components, and heritability estimation of thirteen traits.

	Replication	Family	Variance Components (%)			H^2^
			Replication	Family	Error	
RHBM ^2^	NS	** ^1^	3.74%	82.93%	13.33%	0.723
RTBM	NS	**	0.72%	95.10%	4.18%	0.915
BBM	NS	**	1.43%	89.69%	8.89%	0.819
RHAG	NS	**	0.08%	95.88%	4.04%	0.919
RTAG	NS	**	4.42%	86.06%	9.52%	0.800
RHN	NS	NS	21.51%	0.06%	78.43%	-
RHL	NS	NS	62.27%	17.22%	20.51%	-
FT	NS	NS	5.73%	61.64%	32.63%	0.307
BTN	NS	**	0.74%	95.07%	4.19%	0.915
RDSN	NS	**	1.54%	93.30%	5.15%	0.895
PH	NS	**	0.27%	96.30%	3.43%	0.931
ABM	NS	NS	44.56%	6.35%	49.09%	-
GY	NS	NS	5.30%	17.44%	77.26%	-

^1^ ** Significant at the 0.01 level. NS, nonsignificant. ^2^ RHBM: rhizome biomass; RTBM: root biomass; BBM: belowground biomass; RHAG: rhizome angle; RTAG: root angle; RHN: rhizome number; RHL: rhizome length; FT: flowering time; BTN: basal tiller number; RDSN: rhizome-derived shoot number; PH: plant height; ABM: aboveground biomass; GY: grain yield.

**Table 3:** Individual plants with rhizome biomass (g) selected for constructing BSA pools.

Plants	Individual Rhizome Biomass (g)			
	H1 ^1^	H2	L1	L2
1	82.47	82.47	3.29	8.87
2	77.91	77.91	3.00	8.71
3	73.17	73.17	1.41	8.61
4	69.86	69.86	1.13	6.11
5	67.41	67.41	0.46	5.18
6		59.47		4.68
7		58.91		3.59
8		57.18		3.29
9		57.07		3.00
10		53.40		1.41
11		52.25		1.13
12		51.86		0.46
Mean	74.16	63.41	1.86	4.59

^1^ H1: first-round high-rhizome-biomass bulk; L1: first-round low-rhizome-biomass bulk; H2: second-round high-rhizome-biomass bulk; L2: second-round low-rhizome-biomass bulk.

**Table 4:** Genomic regions of rhizome presence and previous QTL.

Primer	Chr	Location (Mb)	Rhizomatousness	Vegetative Branching
Xtxp43~Xtxp46	1	57.3~80.5	qRZ1.2 [13]; qRN1.2 [13]; Xcup73-Xcup22 [13]; Over-wintering2011B [15]; Ln2011Dist [15]; Ln2010Dist [15]; Ln2010RDS [15]	qTL1.1 [32]; qAX1.1 [32]; qIM1.1 [32]; qVG1.1 [32]
Xtxp471~Xtxp296	2	59.1~70.9		qVG2.1 [32]; qM1_2.1 [32]; qIM2_2.1 [32]
Xtxp26~Xtxp41	4	4.9~59.2	qRZ4.2 [33]	qTL4.1 [32]
Xtxp453~Xtxp123	5	67.1~69.7		
Xtxp40~Xtxp295	7	0.83~62.3	qRZ7.1 [13]; qRN7.1 [13]; pSB067-pSB784 [14]	
Xtxp35~Xgap34	8	55.5~61.8		
Xtxp410~Xtxp287	9	2.1~4.2		qTR9.1 [32]
Xtxp309	10	11.1		

## Data Availability

Dataset available on request from the authors.

## Supplementary Materials

The following supporting information can be downloaded at https://www.mdpi.com/article/10.3390/plants14050713/s1, Table S1. The Basic Statistics and Normality Test for F_3:4_. Table S2. Logarithmic, arctangent and reciprocal transformation of thirteen traits. Table S3. Correlation analysis for F_3:4_ traits. Table S4. SSR markers linked to rhizome presence.

## Abbreviations

The following abbreviations are used in this manuscript:

RHBM	rhizome biomass
RTBM	fibrous root biomass
BBM	belowground biomass
RTAG	root growth angle
RHAG	rhizome growth angle
RHN	rhizome number
RHL	rhizome length
FW	flowering time
BTN	basal tiller number
RDSN	rhizome-derived shoot number
ABM	aboveground biomass
PH	plant height
GY	grain yield
HIF	heterogeneous inbred family
BSA	bulked segregant analysis
SSR	simple sequence repeat
QTL	quantitative trait locus
