# Supplementary material for: Breeding Potential for Increasing Carbon Sequestration via Rhizomatous Grain Sorghum
Source: Plants (Basel). 2025 Feb 26;14(5):713. doi: 10.3390/plants14050713 (PMC11902033; doi:10.3390/plants14050713)
Supplement: Supplementary file 1 [file plants-14-00713-s001.zip › plants-3486983-supplementary.pdf]

plants

MDPI

Article

Breeding Potential for Increasing Carbon Sequestration via Rhizomatous Grain Sorghum

Yaojie Zheng [1], Dirk B. Hays [2,3], Russell W. Jessup [3] and Bo Zhang [1,*]

[1] School of Plant and Environmental Sciences, Virginia Tech, Blacksburg, VA 24061, USA
[2] Texas A&M AgriLife Research and Extension Center at Weslaco, Weslaco, TX 78596, USA
[3] Department of Soil and Crop Sciences, Texas A&M University, College Station, TX 77843, USA
* Correspondence: zhang76@vt.edu

Table S1. The Basic Statistics and Normality Test for F3:4.

Traits [1]	Basic Statistics						Test for Normality	
	BTx623	S. *Propinquum*	F3:4					
	Mean	Mean	Mean	SD	Min	Max	W-value	Pr<W
RHBM (g)			1.80	2.14	0	10.40	0.723	<0.001
RTBM (g)	28.01	52.71	49.38	41.77	14.83	231.26	0.706	<0.001
BBM (g)	28.01	52.71	51.18	41.44	14.83	232.82	0.706	<0.001
GY (g)			5.14	3.89	0	17.75	0.907	0.0027

[1] RHBM: Rhizome Biomass; RTBM: Root Biomass; BBM: Belowground Biomass; GY: Grain Yield.

Table S2. Logarithmic, arctangent and reciprocal transformation of thirteen traits.

Traits[1]	Logarithmic		Arctangent		Reciprocal	
	W-Value	Pr<W	W-value	Pr<W	W-value	Pr<W
RHBM	0.8596	<0.0001	0.5596	<0.0001	0.1167	<0.0001
	0.8876	<0.0001	0.4323	<0.0001	0.3144	<0.0001
RTBM	0.8459	<0.0001	0.4747	<0.0001	0.4130	<0.0001
	0.8976	<0.0001	0.5131	<0.0001	0.4855	<0.0001
BBM	0.9169	<0.0001	0.5373	<0.0001	0.5241	<0.0001
	0.9073	<0.0001	0.4421	<0.0001	0.4299	<0.0001
RHAG	0.9689	0.0268	0.8630	<0.0001	0.8623	<0.0001
	0.9368	0.0007	0.8978	<0.0001	0.8975	<0.0001
RTAG	0.8265	<0.0001	0.6019	<0.0001	0.6011	<0.0001
	0.9428	0.0014	0.9464	0.0022	0.9467	0.0022
RHN	0.9184	<0.0001	0.7079	<0.0001	0.6928	<0.0001
	0.9041	<0.0001	0.4522	<0.0001	0.3759	<0.0001
FT	0.8576	<0.0001	0.8768	<0.0001	0.8765	<0.0001
	0.8667	<0.0001	0.8780	<0.0001	0.8773	<0.0001
BTN	0.9419	0.0004	0.8302	<0.0001	0.7687	<0.0001
	0.8975	<0.0001	0.8421	<0.0001	0.8014	<0.0001
RDSN	0.9455	0.0008	0.4546	<0.0001	0.5732	<0.0001
	0.9502	0.0044	0.5464	<0.0001	0.5962	<0.0001
PH	0.9094	<0.0001	0.8097	<0.0001	0.8090	<0.0001
	0.7592	<0.0001	0.4602	<0.0001	0.4598	<0.0001
GY	0.9700	0.0325	0.4034	<0.0001	0.4015	<0.0001
	0.9636	0.0227	0.5455	<0.0001	0.5449	<0.0001

[1] RHBM: Rhizome Biomass; RTBM: Root Biomass; BBM: Belowground Biomass; RHAG: Rhizome Angle; RTAG: Root Angle; RHN: Rhizome Number: RHL: Rhizome Length; FT: Flowering Time; BTN: Basal Tiller Number; RDSN: Rhizome-Derived-Shoots Number; PH: Plant Height; ABM: Aboveground Biomass; GY: Grain Yield.

Table S3. Correlation analysis for F3:4 traits.

	RHBM	RTBM	BBM	GY
RHBM[1]	1.0000			
RTBM	-0.1791	1.0000		
BBM	-0.1288	0.99871**	1.0000	
GY	-0.0270	0.0985	0.0979	1.0000

[1] RHBM: Rhizome Biomass; RTBM: Root Biomass; BBM: Belowground Biomass; GY: Grain Yield.

Table S4. SSR markers linked to rhizome presence.

Marker	Chr	Location (Mb)	Sequence[1]
Xtxp43	1	57.3	GTTTTCCCAGTCACGAGTCACAGCACACTGCTTGTC<br>CGTCTCGCGGTCCATTTAA
Xtxp433	1	72.3	GTTTTCCCAGTCACGACAAGCGAGATTACAAGGCCCAACCA<br>GCTAGTTAAGAACGTTGACG
Xtxp248	1	79.1	GTTTTCCCAGTCACGACAAGCGGGTGTCCAATGTTGTCTGC<br>ACTCATTCCCTGTCATTGCCGG
Xtxp323	1	79.8	GTTTTCCCAGTCACGACAAGCTATATGCATGTTTTAGGTCG<br>CCTTCTTTCCTTGTTGTC
Xtxp46	1	80.5	GTTTTCCCAGTCACGACAAGCGGGCAATCTTGATGGCGACAT<br>CAAGAGGGGCTCGGTGTGGA
Xtxp471	2	59	GTTTTCCCAGTCACGACAAGCCCGCTTCCTCTCCACTCC<br>TTCTGACCCTTCACCCTCAC
Xtxp296	2	70.9	GTTTTCCCAGTCACGCAGAAATAACATATAATGATGGGGTGAA<br>TTGAGATGTCCGAGATTTAGTATTGTCGTA
Xtxp26	4	4.9	GTTTTCCCAGTCACGACAAGCAAGTGTAGTAGCAGTTTAGTCTC<br>GGAACCAGGAAACTATGGAT
Xtxp41	4	59.2	GTTTTCCCAGTCACGACAAGCTCTGGCCATGACTTATCAC<br>GTTCCCTCAGATGCGGTAAA
Xtxp453	5	67.1	GTTTTCCCAGTCACGACAAGCCGACCTGGAATTGGAATGAA<br>AGATGCGGCTACAACAAGGA
Xtxp123	5	69.7	GTTTTCCCAGTCACGACAAGCTCGGCGAGCATCTTACA<br>TTAGGTTGGCGGATGCAT
Xtxp40	7	0.83	GTTTTCCCAGTCACGACAAGCCAGCAACTTGCACTTGTC<br>GATCACGGTTTAACGAGGG
Xtxp295	7	62.3	GTTTTCCCAGTCACGAAATCATGCATCCATGTTCGTCTTC<br>ATTCGATACTTACATGAGAACATCGCCCTC
Xtxp354	8	55.5	GTTTCCCAGTCACGACAAGCTGGGCAGGGTATCTAACTGA<br>AGTTCCGAGTCTTTTTCCG
Xtxp250	8	58.3	GTTTTCCCAGTCACGACAAGCGCACATCCTCTAAAACTACTTAGT<br>TAGATAGTGTAGCAGGACAAG
Xgap34	8	61.8	GTTTTCCCAGTCACGACAAGCAACAGCAGTAATGCCACAC<br>CTTCTGTTCAAGAGATGGTTCAGT
Xtxp410	9	2.1	GTTTTCCCAGTCACGACAAGCGGCGCCGTATAAAATAGCAA<br>TCTTTTGTTCCTGCGGGAGA
Xgap42	9	3.6	GTTTTCCCAGTCACGTTTTCCTCTTTCAGATAACCGTA<br>CTACGGGAACCACCC
Xtxp287	9	4.2	GTTTTCCCAGTCACGGCAAGCGAGCTGACTTATGTAACGAGA<br>AAGTGGGACGTATCCAAATCATCGTGAAAC
Xtxp309	10	11.1	GTTTTCCCAGTCACGACAAGCTGCCCTTCAGGAATGATTCGAC-<br>TACTAC<br>AAAAAGAGGATAAACACCGTAAAACGT

[1] The forward and reverse marker sequence were from -5′~ -3.′
